# Using self-reported height and weight in calculating body surface area: is it good practice in cardiovascular imaging?

**DOI:** 10.1186/1532-429X-14-S1-P39

**Published:** 2012-02-01

**Authors:** Emer Sonnex, Kate Mracek, Melissa L Grzeszczak, Richard Coulden

**Affiliations:** 1 Diagnostic Imaging, University of Alberta Hospitals, Edmonton, AB, Canada

## Summary

Cardiac MR measurements of mass and volumes are normalized to patient height and weight. Many centers use self-reported BSA. This practice is easy and quick but is it accurate enough in this setting? We compared measured height and weight against self-reported figures, sub-dividing the data sets into sex and age to see if any group was more reliable in their self-reporting.

## Background

Cardiac dimensions and volumes are widely used throughout cardiac imaging. Body mass index (BMI) and body surface area (BSA) can both be used but BSA is most validated. There are many formulae for calculating BSA but the Mosteller calculation is the easiest, can be performed on a basic calculator and has gained in popularity. BSA = ((height cm*weight kg)<-2>/3600)). Many centers use self-reported height and weight for BSA calculation and subsequent normalization. Is this reliable?

## Methods

All patients attending for cardiac MRI complete a safety questionnaire which also asks for self-reported height and weight. All cardiac MRI patients are also weighed and height measured (stocking feet) after changing into a hospital gown. We reviewed the height and weight of 238 patients over 4 months (97 females; 141 males; mean age 42 years, 18stdev). We compared the self-reported values to those measured.

## Results

67% of patients mis-reported their weight by 1kg or more (male 73%; females 58%), p< 0.001 while 69% patients mis-reported their height by 1.25cms or more (68% males; 70% females), p < 0.001. Half (50%) of women and 37% of men under-estimated their weight while 66% of women and 51% of men under-estimated their height. Although 1 woman (76 years old) weighed 6.4kgs less than she reported, 28 women (29%) weighed 2kgs or more than they self-reported with 10 (10%) weighing 4 kgs or more (range 4 - 12.3kgs). Of these 10 patients, 7 were in the 40 - 50 year age group. Interestingly 4 women thought they were taller (>1.25cms) and 58 (60%) thought they were shorter. 15 men weighed 2kgs or less (range 2 - 15.4) than they reported, 47 men (33%) weighed 2 kgs more than their self-reported weight with 21 (15%) weighing more than 4kgs (range 4 - 24.9kgs). Again, the largest group (34%) was in the 40 - 50 year age group. 56 men (40%) were 2.5cms (range 2.5 - 7cms) shorter than they reported, 8 (6%) were actually taller than they reported (range 2.5 - 6.5cms).

## Conclusions

Published review articles have indicated that women underestimate their weight and overestimate their height while older women are less reliable in their self-reported BSA. Men have also been reported to frequently overestimate their height. In our group, there was no set pattern and both men and women were inaccurate in their self-reported heights and weights. This was true for all age groups. Self-reported heights and weights are inaccurate with no clear age or sex pattern. Although it adds another step in the patient journey through the MRI department, all patients should have their height and weight measured before their cardiac MRI examination.

## Funding

Not applicable.

**Table 1:** Self-reported (SR) compared to measured (M) heights and weights

Total = 238	Age	SR weight (kgs)	M weight (kgs)	SR height (cms)	M height (cms)
mean	42	79.91	81.57	168.45	170.43
stdev	18	24.87	25.49	11.1	10.94
minimum	6	23.18	23.1	117.50	118
maximum	85	171.82	169	192.5	197

female = 97					

female mean	38	67.99	69.51	160.34	162.81
female stdev	18	21.4	21.89	7.39	7.95
female minimum	10	28.18	28.1	140	139
female maximum	85	154.55	157.5	180	182

male = 141					

male mean	45	88.11	89.86	174.04	175.67
male stdev	19	23.81	24.52	9.69	9.57
male minimum	6	23.18	23.1	117.5	118
male maximum	79	171.82	169	192.5	197

